# Supplementary material for: Targeting transcription factors through an IMiD independent zinc finger domain
Source: EMBO Mol Med. 2025 May 14;17(6):1393–416. doi: 10.1038/s44321-025-00241-3 (PMC12163085; doi:10.1038/s44321-025-00241-3)
Supplement: Supplementary file 1 — Appendix [file 44321_2025_241_MOESM1_ESM.pdf]

Appendix

Table of Contents

Appendix Figure S1 .... **2**

Appendix Figure S2 .... **3**

Appendix Figure S3 .... **4**

Appendix Reference **5**

Appendix Figure S1

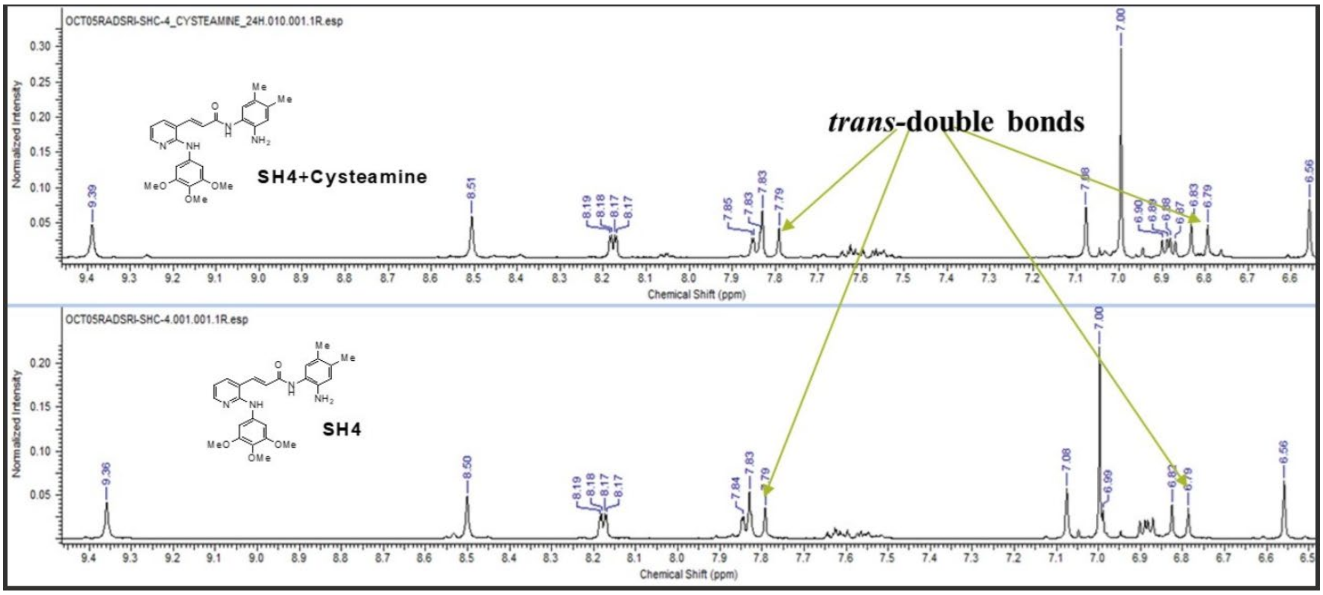

Appendix Figure S1. ^{1}H NMR spectrum of the acrylamide double bond. The ^{1}H NMR spectrum of SH4 (0.05 mmol) was recorded in DMSO-d6 (0.5 mL). To the same sample, in a tightly capped Eppendorf tube, cysteamine (0.5 mmol, Sigma-Aldrich, USA) was added, sonicated for 10 min, and quickly transferred to an NMR tube for the recording of the spectrum. The ^{1}H NMR spectrum of the same sample was collected again after 24 hours standing at room temperature. ^{1}H NMR spectra were analyzed using Mnova 10.0.2 (Mestrelab Research, CA, USA).

Appendix Figure S2

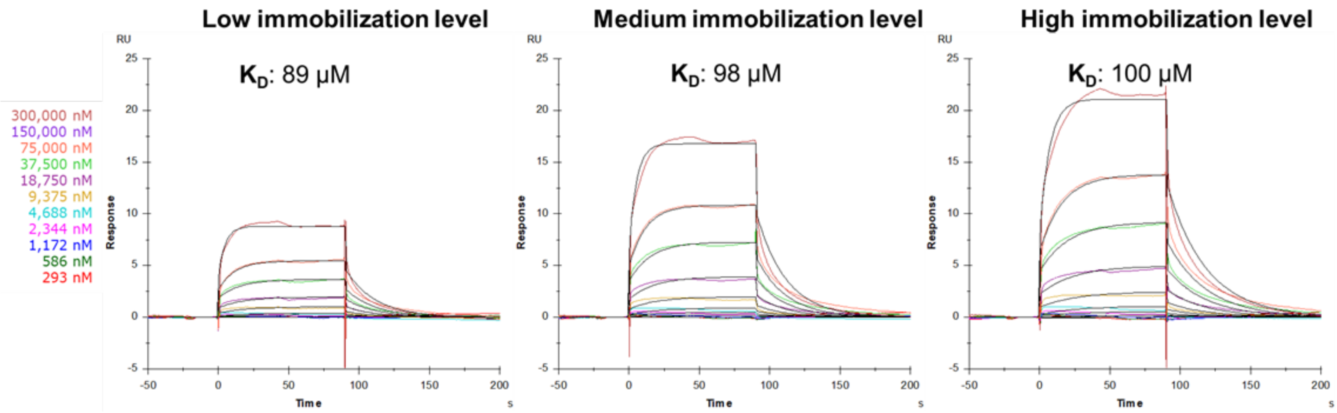

Appendix Figure S2. SPR binding data with 1:1 modelling for the interaction between CUL4A and SH6. The same interaction was tested using 3 different immobilization levels for CUL4A: low (1,700 RU); medium (2,800 RU); and high (4,000 RU). Each plot shows a 2-fold dilution series of SH6 compound, with the top concentration 300 µM.

Appendix Figure S3

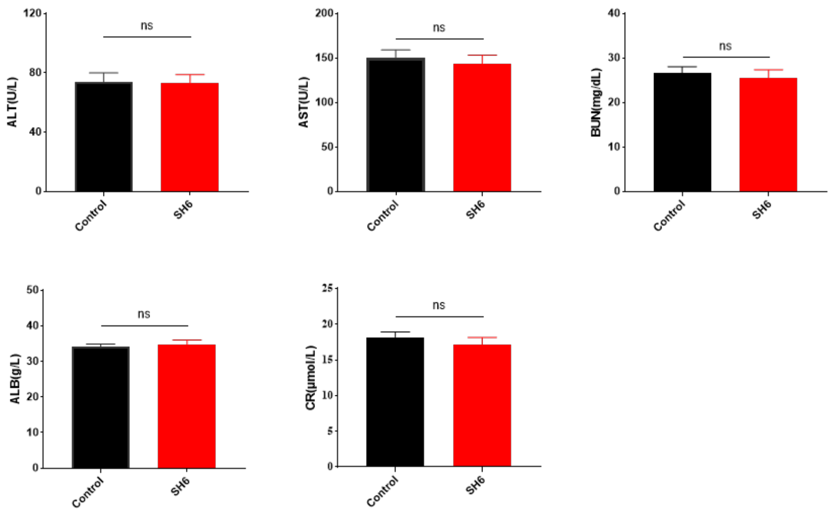

Appendix Figure S3. For the PDX model, biochemistry studies of the liver (alanine transaminase (ALT), aspartate aminotransferase) (AST), Albumin (ALB)), and renal (Blood urea nitrogen (BUN) and creatinine (CR)) functions were examined by collecting mouse peripheral blood at the end of treatment. No significant change between the two groups (control and SH6 treated) was observed. N.S., not significant. n= number of mice (n=8). Error bars = standard error of the mean (SEM). *P* values were calculated by the two-tailed Student's t-test.

Appendix Reference for Tandem Mass Tag Mass Spectrometry (Dataset EV6)

1. Padmanabhan N, Kyon HK, Boot A, Lim K, Srivastava S, Chen S, Wu Z, Lee HO, Mukundan VT, Chan C, Chan YK, Xuewen O, Pitt JJ, Isa ZFA, Xing M, Lee MH, Tan ALK, Ting SHW, Luftig MA, Kappei D, Kruger WD, Bian J, Ho YS, Teh M, Rozen SG, Tan P. Highly recurrent CBS epimutations in gastric cancer CpG island methylator phenotypes and inflammation. Genome Biol. 2021 Jun 1;22(1):167. doi: 10.1186/s13059-021-02375-2. Erratum in: Genome Biol. 2021 Jun 17;22(1):181. PMID: 34074348; PMCID: PMC8170989.
2. Cox J, Hein MY, Luber CA, Paron I, Nagaraj N, Mann M. Accurate proteome-wide label-free quantification by delayed normalization and maximal peptide ratio extraction, termed MaxLFQ. Mol Cell Proteomic. 2014;13(9):2513–2526. doi: 10.1074/mcp.M113.031591.
