# Supplementary figures and images for: Targeting transcription factors through an IMiD independent zinc finger domain
Source: EMBO Mol Med. 2025 May 14;17(6):1393–416. doi: 10.1038/s44321-025-00241-3 (PMC12163085; doi:10.1038/s44321-025-00241-3)

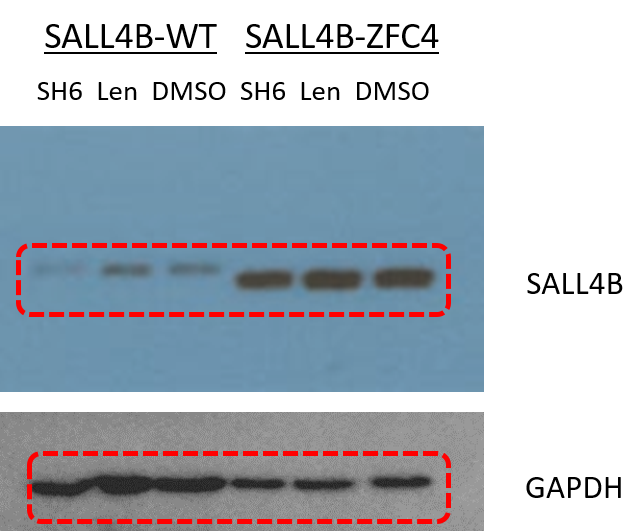

Supplement: Supplementary file 11 — Source data Fig. 4 [file 44321_2025_241_MOESM11_ESM.zip › Figure 4/Fig 4A.png]

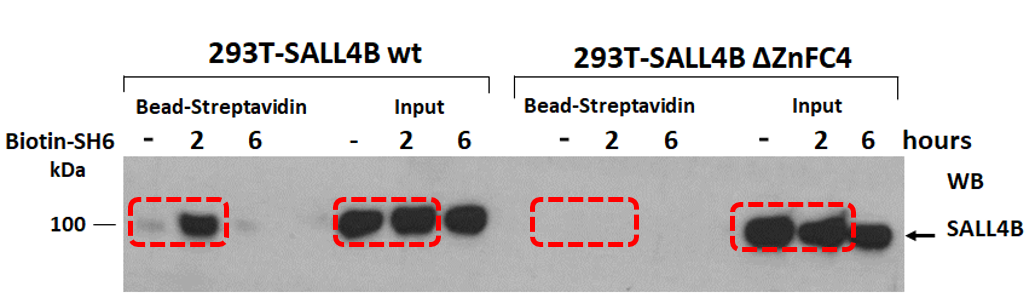

Supplement: Supplementary file 11 — Source data Fig. 4 [file 44321_2025_241_MOESM11_ESM.zip › Figure 4/Fig 4B.png]

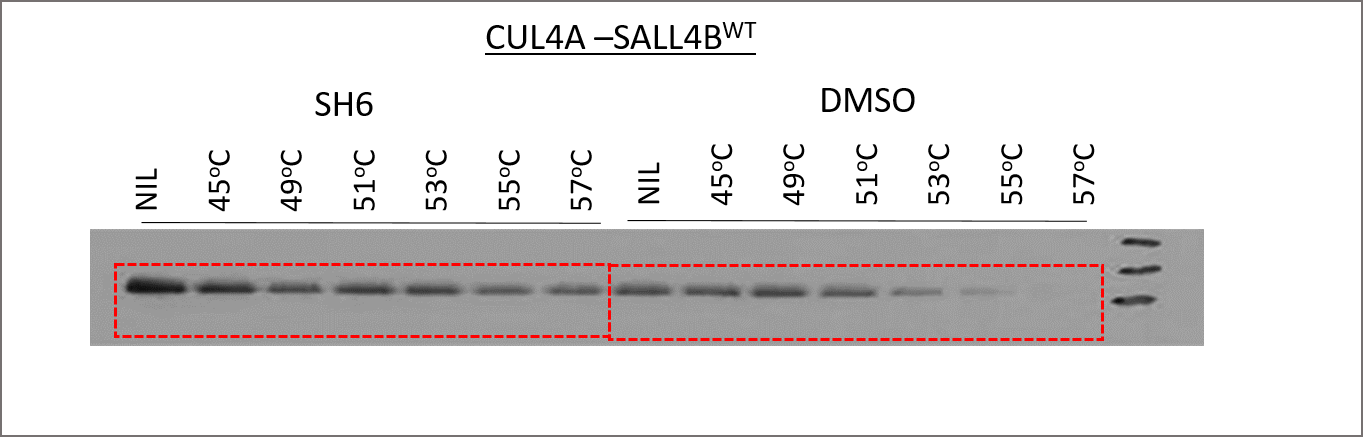

Supplement: Supplementary file 11 — Source data Fig. 4 [file 44321_2025_241_MOESM11_ESM.zip › Figure 4/FIg 4C(i).png]

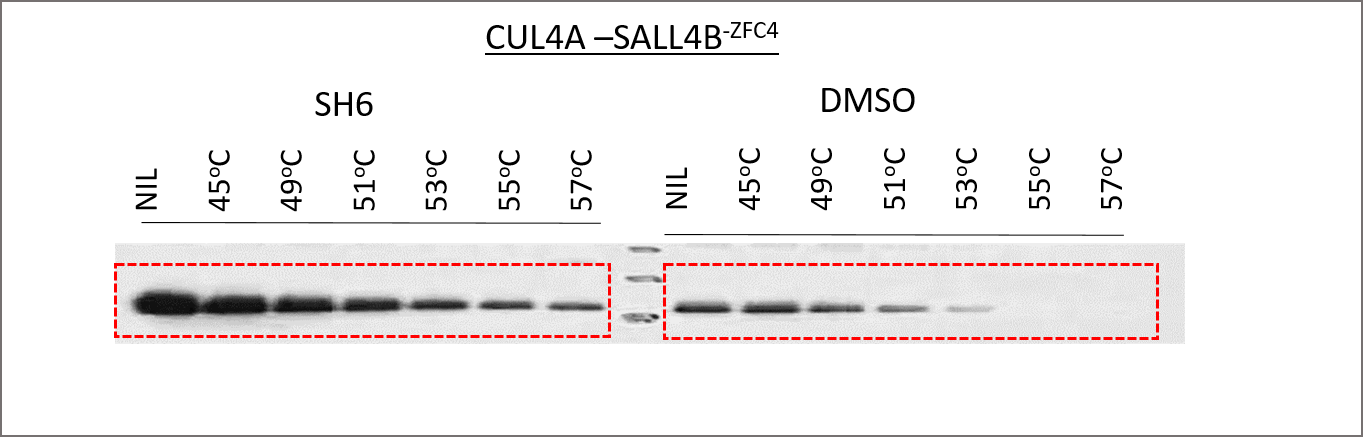

Supplement: Supplementary file 11 — Source data Fig. 4 [file 44321_2025_241_MOESM11_ESM.zip › Figure 4/FIg 4C(ii).png]

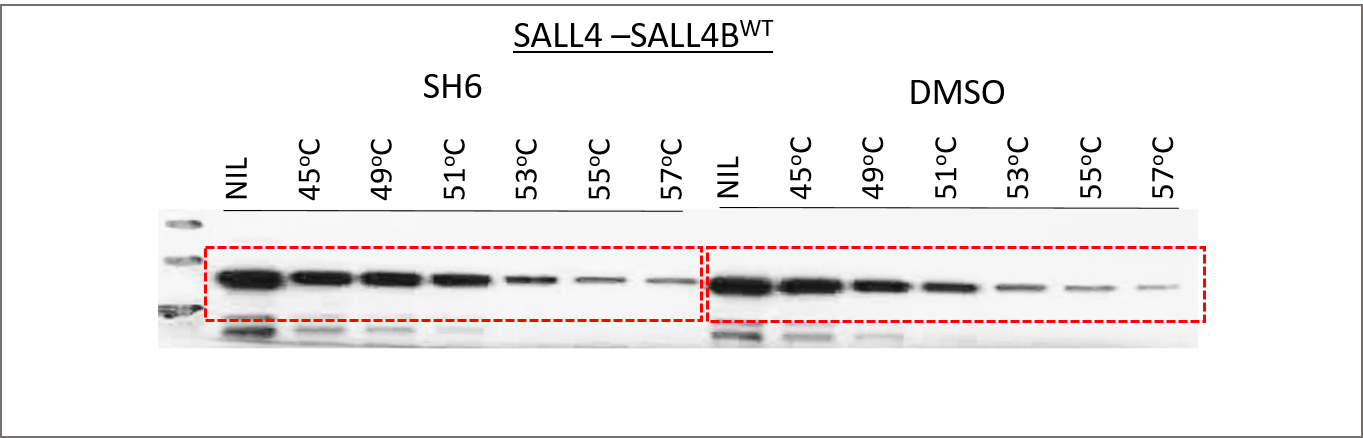

Supplement: Supplementary file 11 — Source data Fig. 4 [file 44321_2025_241_MOESM11_ESM.zip › Figure 4/FIg 4C(iii).png]

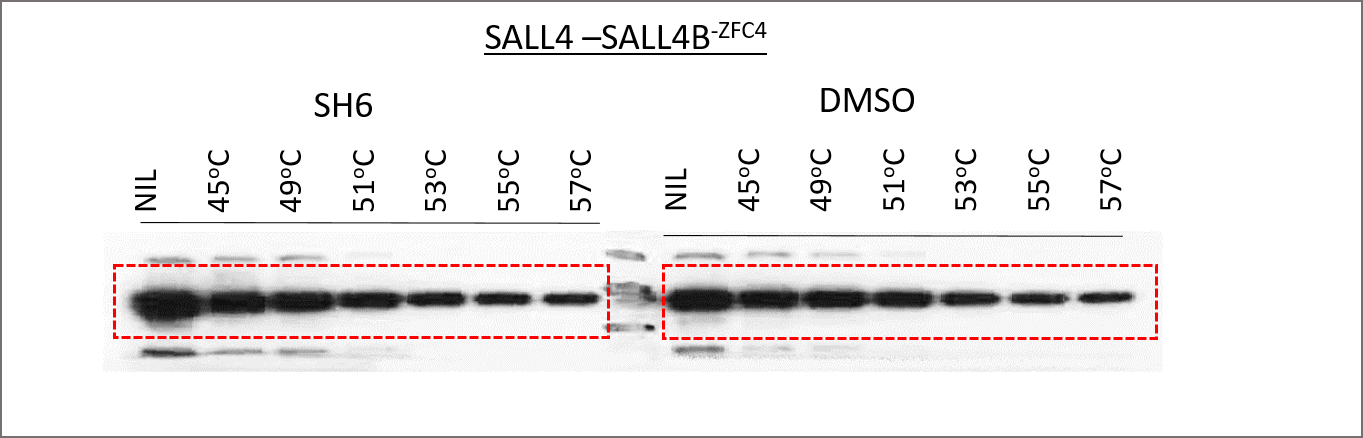

Supplement: Supplementary file 11 — Source data Fig. 4 [file 44321_2025_241_MOESM11_ESM.zip › Figure 4/FIg 4C(iv).png]

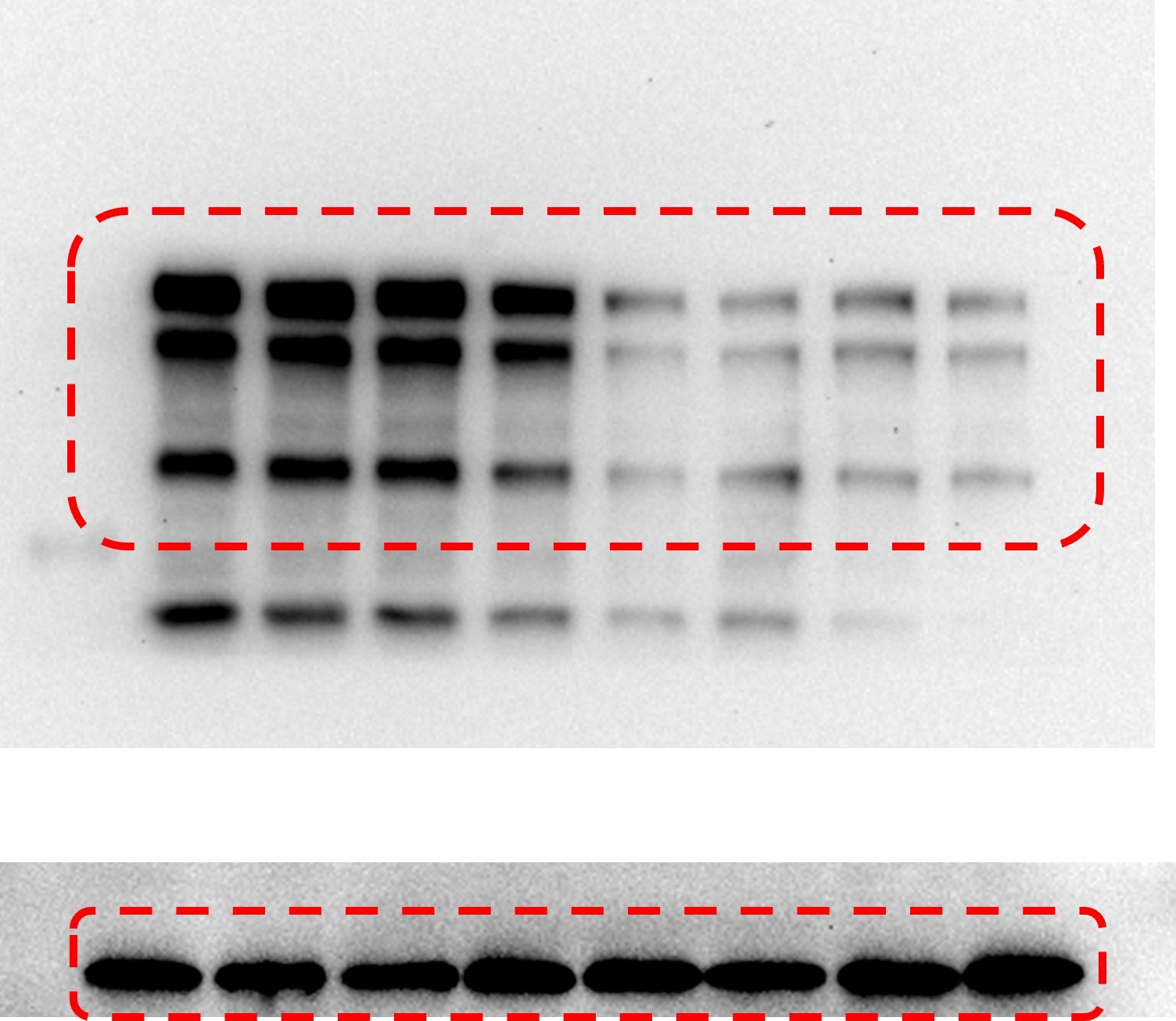

Supplement: Supplementary file 12 — Source data Fig. 5 [file 44321_2025_241_MOESM12_ESM.zip › Figure 5/Fig 5C (iii).png]

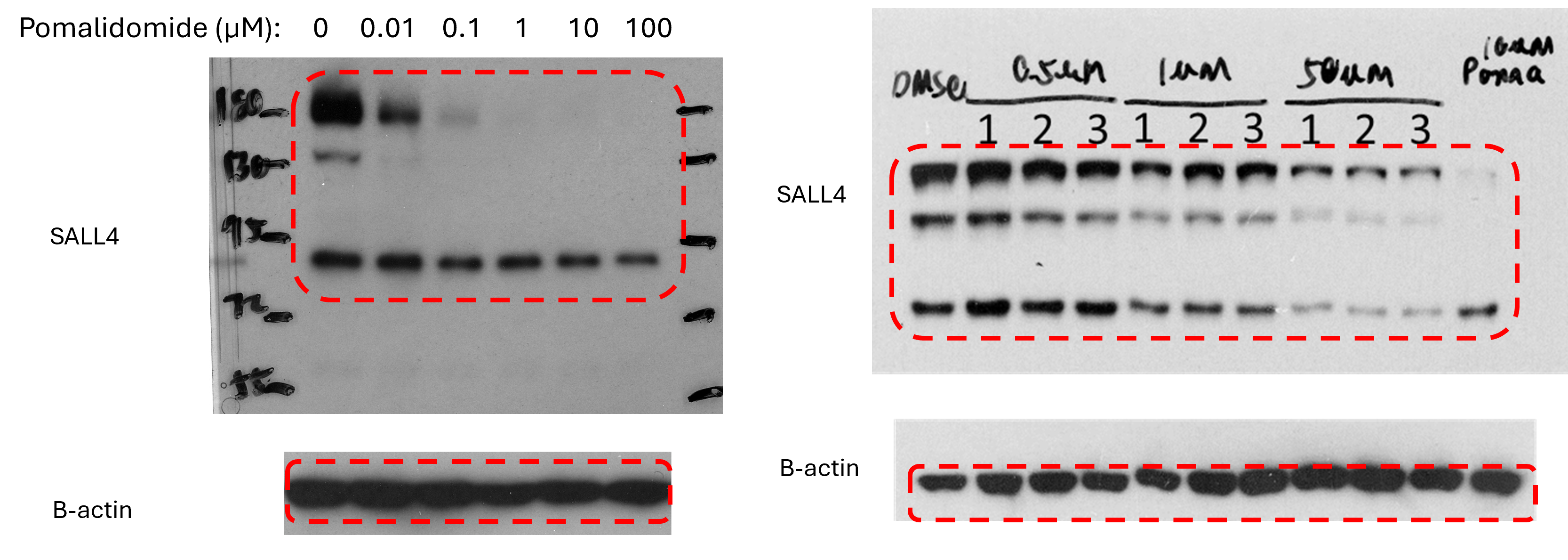

Supplement: Supplementary file 13 — Source data Fig. 3 [file 44321_2025_241_MOESM13_ESM.zip › Figure 3/Fig 3A.png]

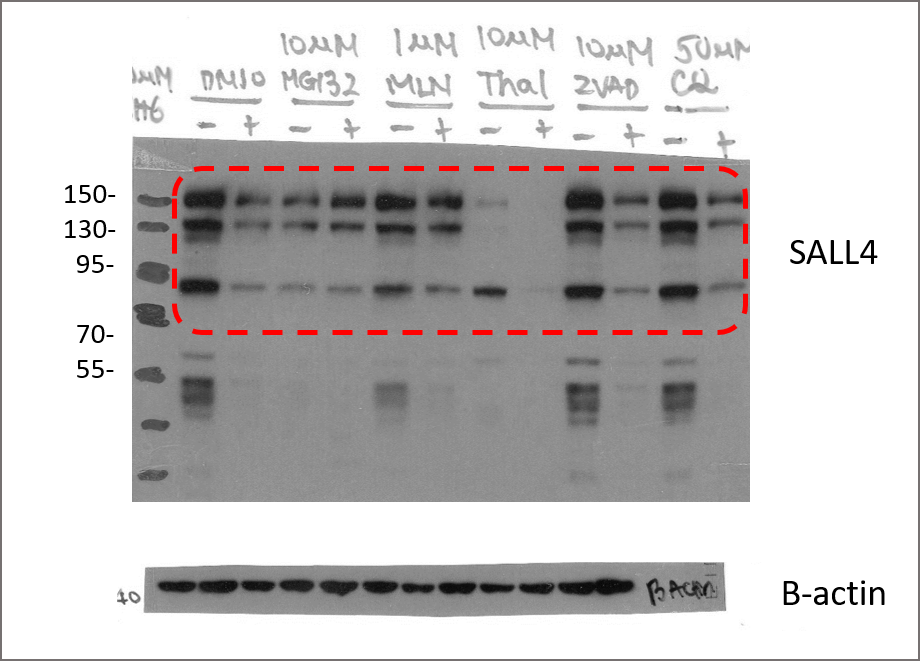

Supplement: Supplementary file 13 — Source data Fig. 3 [file 44321_2025_241_MOESM13_ESM.zip › Figure 3/Fig 3D.png]

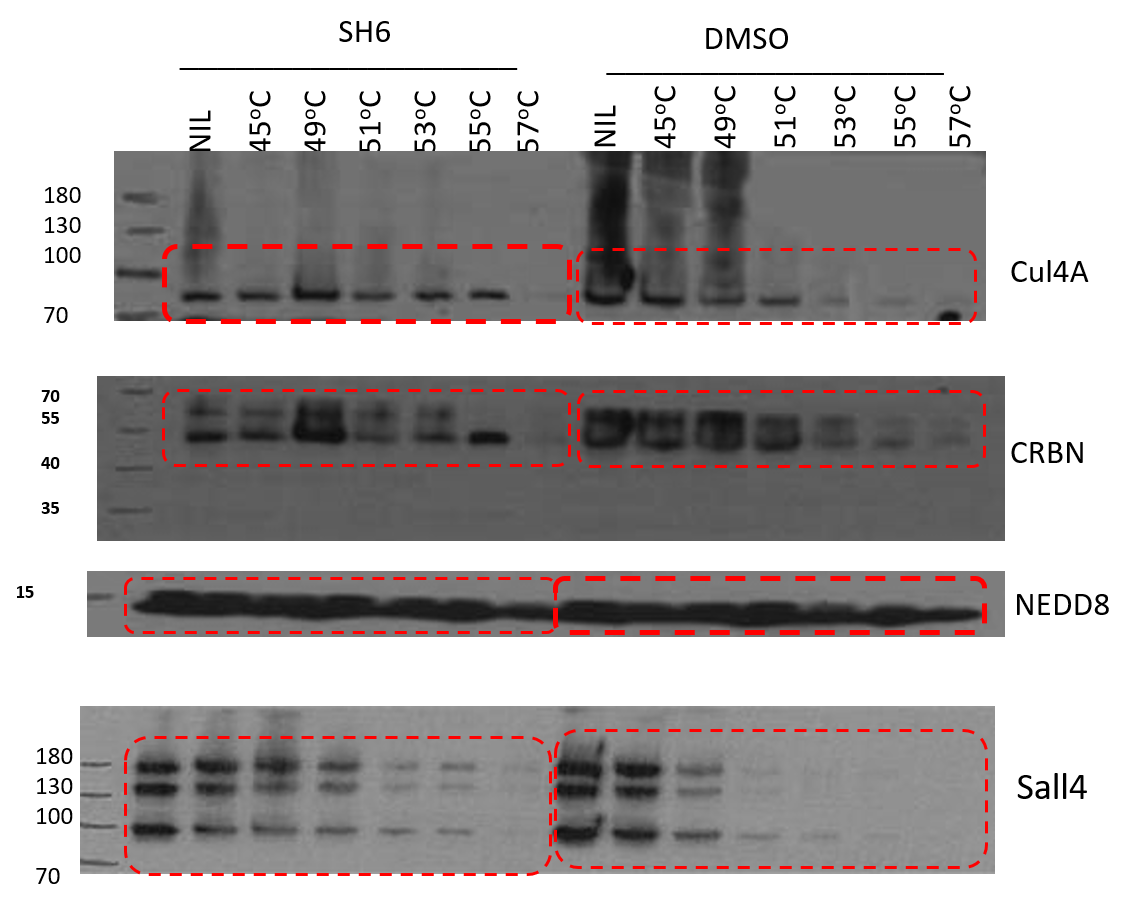

Supplement: Supplementary file 13 — Source data Fig. 3 [file 44321_2025_241_MOESM13_ESM.zip › Figure 3/Fig 3F.png]

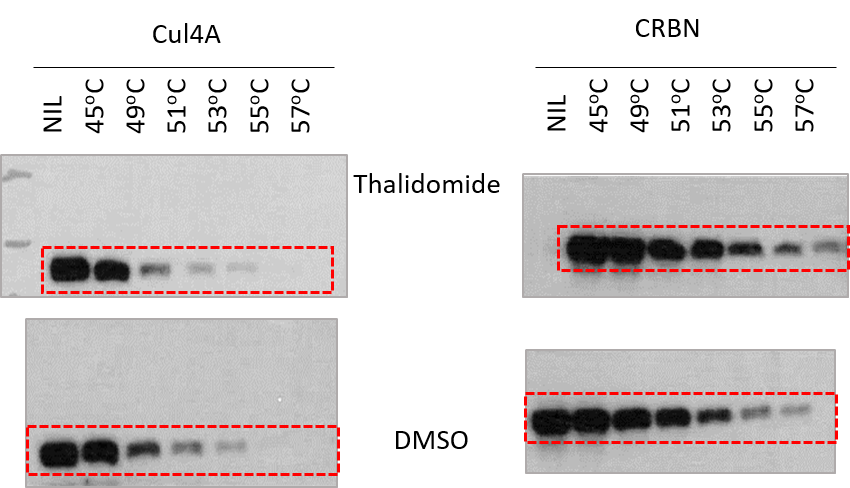

Supplement: Supplementary file 13 — Source data Fig. 3 [file 44321_2025_241_MOESM13_ESM.zip › Figure 3/Fig 3G.png]
