# Supplementary material for: Targeting transcription factors through an IMiD independent zinc finger domain
Source: EMBO Mol Med. 2025 May 14;17(6):1393–416. doi: 10.1038/s44321-025-00241-3 (PMC12163085; doi:10.1038/s44321-025-00241-3)
Supplement: Supplementary file 14 — Expanded View Figures [file 44321_2025_241_MOESM14_ESM.pdf]

Expanded View Figures

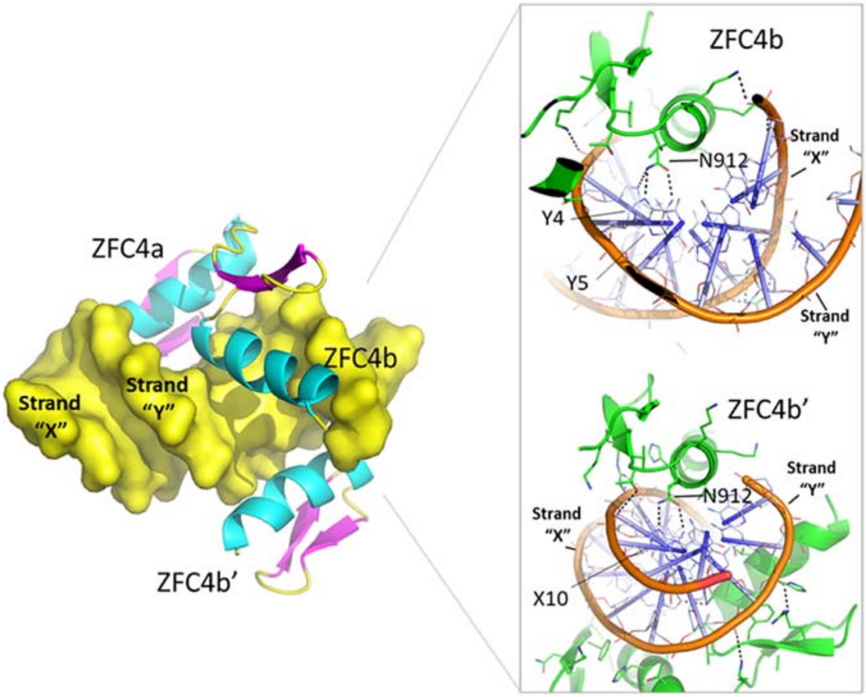

Figure EV1. Crystal Structure of the fourth SALL4 zinc finger cluster (ZFC4, teal and magenta) bound to DNA (yellow).

The structural view (left) demonstrates how the two zinc fingers of the first SALL4 molecule (ZFC4b & ZFC4b′) and the second zinc finger of the second SALL4 molecule (ZFC4a) in the asymmetric unit bind in the major groove of DNA. Structural view in the box (right) depicting the specific interactions between N912 of SALL4 and adenines at position Y4, Y5, and X10 of DNA.

 © The Author(s)

A

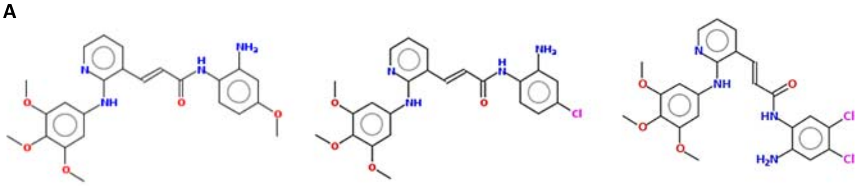

SH2 SH6 SH7

	SH2	SH6	SH7
cLog P [a]	2.60	3.54	4.25
PAMPA Effective Permeability Pe (10^{-6}cm/s) pH7.4 [b]	-	66.1 (±6.8)	41.2 (±5.1)

B

	Avg P (10^{6}cm/s) , pH 7.4	SD	Classification
Antipyrine	1.28	0.57	Low
Carbamazepine	65.22	0.37	High

Figure EV2. Structures and physicochemical properties of potent analogs SH2, SH6, and SH7.

(**A**) These compounds have the lowest EC50 values (<3 μM) on SALL4 high SNU 398 cells. (**B**) Permeability test (PAMPA) result of the negative control (Antipyrine) and positive control (Carbamazepine).

© The Author(s)

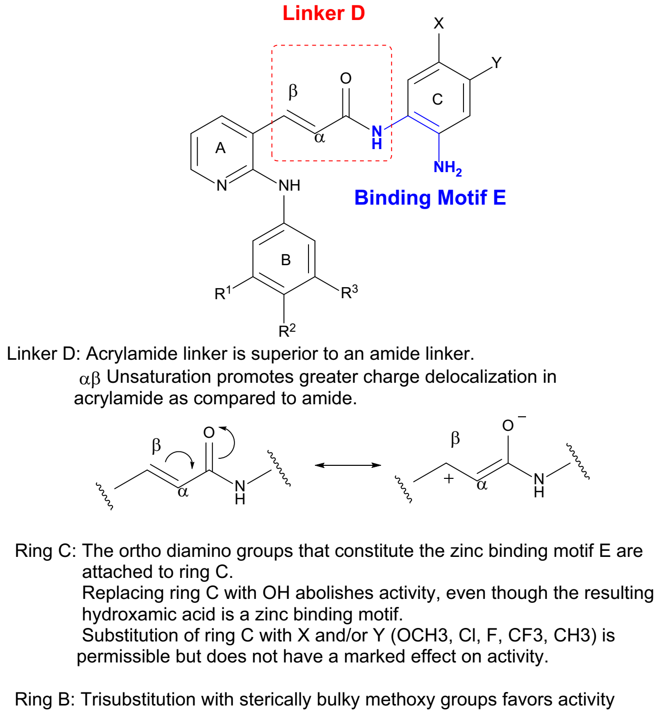

Figure EV3. Structural activity of SH library.

Phenotypic screening of the SH library on SALL4 cell lines (Dataset EV4) revealed three key structure-activity correlations. First, the acrylamide linker D is an indispensable feature for activity. Replacing this linker with an amide resulted in compounds which have markedly lower activity against the SALL4 high cells. In fact, of the compounds with amide linkers (scaffold B-2, Fig. 1H), only four (SH43,47,69,71) have EC50 values ≤20 μM on SALL4 high cells, as compared to 31 out of 35 compounds in acrylamide bearing scaffold B-1. Unlike the amide, electron withdrawal by the carbonyl oxygen in the acrylamide linker delocalizes the positive charge onto the β carbon, resulting in more extensive distribution of charge in the acryan electron deficient β carbon which is susceptible to reaction with electron rich species. It is conceivable that this enhanced reactivity contributed to the greater cell-based potencies of the acrylamide-based SH compounds. A second requirement for potent activity is the ortho diamino motif E derived from the HDAC component in the hybrid scaffold. Of the two ortho-diamino groups, one is embedded within the acrylamide linker D while the other is a substituent on ring C. The ortho positioning of the amino groups is optimal for zinc binding. When Ring C is replaced by hydroxyl (OH) as seen in scaffold B-3 (SH8, SH16, SH24, SH32, SH40), activity was diminished, notwithstanding the retention of the zinc binding motif which is now represented by hydroxamic acid. The diminished activity is likely due to the absence of the sterically larger and bulkier ring C. Lastly, we noted that 6 of the 8 potent compounds (EC50 < 3 μM, all from scaffold B-1) have a trimethoxy substituted ring B. Rings A and B are part of the scaffold found in the antimicrotubule agent E7010, with ring B attached to the ortho position of pyridyl ring A. Mono-substitution of ring B with either methoxy, chloro, fluoro, or trifluoromethyl generally diminished activity. Possibly the sterically bulky and lipophilic trimethoxy substituted ring B has a role in reinforcing interactions with the putative receptor, hence contributing to enhanced potency.

 © The Author(s)

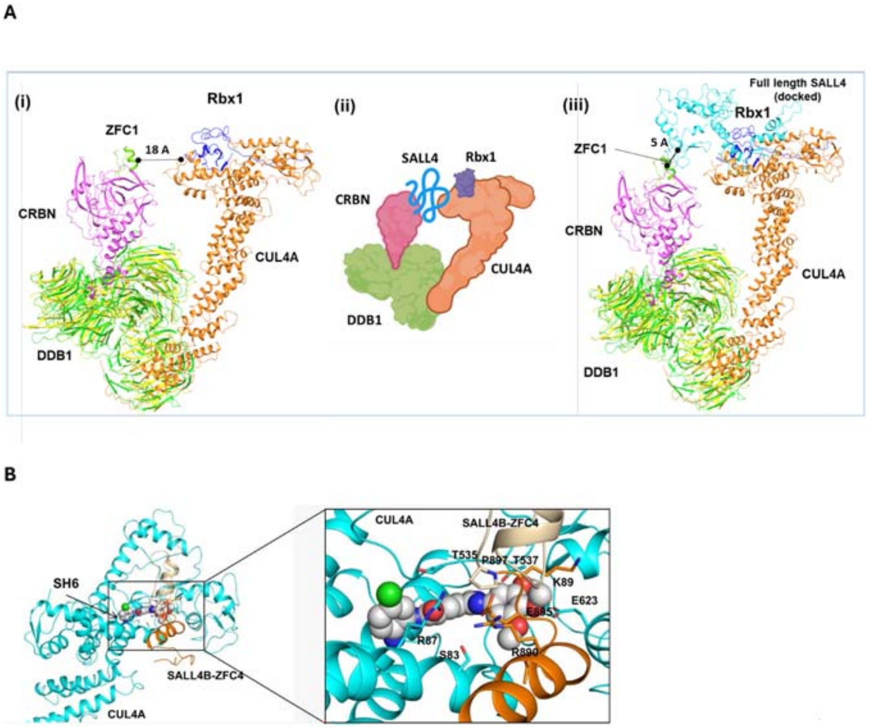

Figure EV4. The structural model of SALL4A recruited to the CRBN-DDB1-CUL4A-Rbx1 complex.

(**A**) ZFC2-4 was predicted to bind to CUL4A near the Rbx1 site. (i) Superimposed 3D structures of the SALL4A ZnF3-CRBN-DDB1(green) complex (PDB ID: 6UML) and DDB1(yellow)-CUL4A-Rbx1 complex (PDB ID: 2HYE) with reference to DDB1. (ii) Cartoon illustration of the SALL4A/B-CRBN-DDB1-CUL4A-RbX1 protein interaction network. (iii) Superimposed 3D structures of the SALL4A ZnF3-CRBN-DDB1(green) complex (PDB ID: 6UML) and DDB1(yellow)-CUL4A-Rbx1-ZFC 2–4 of the SALL4A complex (generated by protein–protein docking) with reference to the DDB1 domain. (**B**) The docking pose of SH6 at the predicted SALL4B ZFC4-CUL4A interface. Wheat and orange = SALL4B ZFC4; Cyan = CUL4A.

© The Author(s)

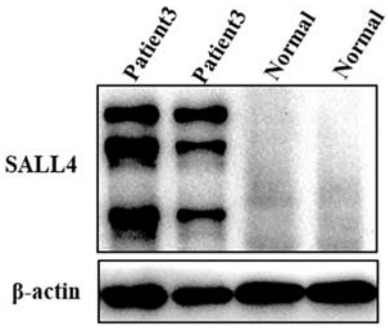

Figure EV5. Western blot analysis of SALL4 protein expression of patient samples.

The PDX used in xenograph studies was established from patient 3 with a high level of SALL4 expression. Normal represents adjacent patient sample which did not express detectable levels of SALL4.

 © The Author(s)
